# On Boys

**Published:** 1870-01

**Authors:** 


### ON BOYS.

BY UNCLE BOB.

I always feel sorry for one half the folks in the world—that half which never was and never can be—*boys*. Yes 'twas nice to be a girl but so, so nice to be a boy, a regular boy-boy.

I don't think much of your pretty, quiet, effeminate boy; no I abominate him, and I have not much faith in your proverbially good boy as a whole,—the boy who is always up to the head in school, who never does anything naughty, who is never found out it he does. I'll tell you the boy I like: the happy, healthy, active, bold boy; restless, truthful and pure minded, who makes his mistakes because he will *do* something, just as fire *will burn* if you put anything in its way.

The boy who climbs fences and falls off them, but don't cry; who climbs trees, tears his trowsers, takes his bumps and bruises, and comes home for repairs preparatory to new adventures; who goes to bed "in ordinary" while the only pair is being "reinforced" as the tailors call it.

Do you suppose that boy goes to sleep when he goes to bed? No he is panting for more. Of course he believes the injunction to be careful, is a good one, but just so sure as he experiences a call to some new adventure the pants must come in for their share of responsibility in the undertaking. "Try again" is written all over him.

Now I don't let down the bars of goodness in tending that all the boys in creation shall come rioting through them, thinking I don't care what a boy does. Remember I put in the words truthful and pure-minded in the description of my boy. Believing as I do in boys, I want others to do so too. I want to do boys a good turn, put myself into sympathy with them and have others see them as I do. I don't want the sour, peevish, idle, reckless boy, the deceitful and untrustworthy boy, who seeks to conceal his mistakes because he is afraid of a punishment, to come into my class of boys. See how my boy takes a correction for an error. He knows he has done wrong and the punishment is just; he does not sulk or snivel—he makes up his mind to endure bravely and look out in future. He settles the matter then and there and is really grateful if he is forgiven after he is punished. He don't want to be reproved, for he has the wit to know that all such talk is buncombe. I say as he says, punish him, kindly, and then forgive him. Don't hold on to him to see if tears cannot be brought into his eyes. He don't believe in tears, they are girlish and isn't he a *boy?* He will begin to sour if he is not dismissed soon after he has confessed. Let him go I say, and I tell you to say to him kindly, and with earnest frankness, to dismiss the scene from his mind, except as a lesson for future caution.

I tell you who ever you are who read this—that boys who are boys won't forget kindnesses. They know what is their due and accept the consequences of their mistakes just as readily as a grown man. Treat the boy as a part of yourself and he will never get abused.

They are not merely restless, noisy shreds of humanity, a trouble and that only. They are not to be pushed out of sight till they can stand up in a fair and square tussle with the world. Give them plenty of beef and pudding, birch and arithmetic in proper proportion, season all this with good advice and then watch them grow. No danger that they will run to wild. I am ready to guarantee that they will call no name so dear as the home they grew up in.

"Heaven lies about us in our infancy"—we are told, yes, all so, but what is to be said of the other meaning of this sentence. Who does the lying afterwards? I say Heavens! what lying is done about boys. Now I like Dickens because he takes so many boys into his stories. He is not afraid to see good in boys. His characters grow right up into the sympathy of every one. I don't like to hear people talk about how much of this sympathy they have unless they take right hold and show it out. I say take an interest in the boys you see,—dare some day to touch a boy on the arm and ask him all about his feelings. Ask him where he lives, what he does, who is his father, if he has any pets, who feeds them, (if he has any.) In short, get right down into that boys soul and thoughts. Make him believe you are interested in him, and he will tell you all he knows and twenty things besides. 'Tis wonderful to see how a real boy will unfold his whole self to any one whom he believes is his friend. Take heed of the way in which he talks to you. See, there is not breath enough in his body to get his words out as he wants to crowd them. Watch the turn of his eye as he sticks at some word to carry a mountain of meaning for his young ideas. That's a *boy* and you know it, and will feel glad all day that you touched him.

I want to take all the world by the hand and say to them as earnestly as I can, *don't snub the boys.* Let them grow up in a belief that the years of their child-life are a protection simply of their size, not of their hearts. Give all place to boys, my boys as I call them, and you shall find them crowding the entire earth for room to show themselves.

